# Between dissonance and desire-queer(ing) adaptive rituals of conformity: wit(h)nessing inequities and social justice in healthcare education in the Global South through body pedagogies

**DOI:** 10.3389/fpubh.2026.1830881

**Published:** 2026-06-05

**Authors:** Mark A. Vicars, Renaissan Dutta, Emilie Das, Shuvojit Moulik

**Affiliations:** 1 ISILC, CoABLIT, Victoria University, Melbourne, VIC, Australia; 2 CWF Labs, Transdisciplinary Healthcare and Research, Bolpur, West Bengal, India; 3 Civilian Welfare Foundation, Kolkata, West Bengal, India; 4 Santosh Deemed to be University, Ghaziabad, India

**Keywords:** body pedagogies, Global South, health care education, LGBTQIA+, wit(h)nessing

## Abstract

The state of healthcare education in the Global South is at a critical point of change. In general terms, the introduction of LGBTQIA+ health content into curricula is widely accepted as an important contribution and a step toward acceptance of gender diversity and inclusion. However, the actual way of teaching this content oftentimes simply continues to perpetuate violence against LGBTQIA+ people and, by extension, their communities. In other words, the historical patterns of teaching LGBTQIA+ people as “problems” (i.e., as pathological entities that need intervention), as at-risk groups that need to be managed, and as an example of how not to be healthy are continued by most educators despite the existence of LGBTQIA+ health content. The way that LGBTQIA+ health content is taught in the Global South reflects the influence of historical power relations that are built into medical education systems inherited from colonial powers as allied health practitioners and medical institutions continue to control whose bodies will be seen as worthy, acceptable, and validated as being a part of the “normal” world and as a result who will have access to health care services. There is limited attention to how LGBTQIA+ body pedagogies can serve as counter-practices to reimagine care, learning, and inclusion in the contexts outlined previously. This study analyzes how body pedagogies can disorient reductionist and interventionist approaches to LGBTQIA+ identities in health care education in the Global South.

## Introduction

This study focuses on how contemporary healthcare education in the Global South, within the geo-cultural and social context of India, is failing to recognize the lived experiences and embodied wisdom of LGBTQIA+ communities. A Western diagnostic paradigm that informs medical praxis in the Global South systemically pathologizes and perpetuates the epistemicide of indigenous, dissident genders and sexualities. Hijra- a Hindi word assigned to India’s “third gender,” Chakka -a derogatory Hindi term used to insult effeminate men or transgender individuals implying deviance or emasculation (1) and Launda –which refers to young men or boys who perform feminized roles in traditional dance performances occupy a culturally specific space that neither straightforwardly ‘transgender’ nor ‘homosexual’ in the Western sense but rather a socially and performatively defined role rooted in the folk practices (2).

Homosexual and genderqueer bodies in the Indian context have a long history of cultural narration and have been burdened with layers of stigma, insult, and historical marginalization. Stereotypes and shame around gender non-conformity and non-heteronormative behavior have, in the Indian context, been connected to the performativity of the “default human” body (3). The “default human” body is men and cisgender, and the privilege it affords highlights the systemic gaps of power hierarchies in medical systems of who gets counted and who does not.

The “default human” body produces practices of exclusion, not as an accident but as an intended outcome of the social and power dynamics built upon interventionism, which we suggest, has orientated the medical gaze to objectifying LGBTQIA+ bodies and subjecting them to an increased doxa of standardization, surveillance, docility, and as subordinate, marked and problematic (4). In the medical field, heterosexuality is seen as the Human Default (unmarked); heterosexual individuals are viewed in a complex manner, i.e., as having complex subjectivity(transcendence), whereas all other sexualities/genders require additional “modules” of study, additional attention, and will always be seen as “Other”.

Medical education serves as a training ground for practitioners to learn which bodies are classified as healthy vs. pathological. In unpacking how medical systems and medical practitioners in clinical observation embed bias and reproduce exclusion derived from being positioned by privileging hegemonic social discourses and normative identity categories, and conduct derived from the bio-power of cisgender privilege.

This study presents a framework for the inclusion of LGBTQIA+ individuals in health care settings that goes beyond tokenistic models of diversity. It considers how health-related issues can be taught and transformed in ways that do not situate LGBTQIA+ individuals as problems in need of solutions but rather as holders of valid expertise based on their experience of navigating through spaces not designed for them. This study draws on the notion of epistemicide (5) and is cognizant that frameworks derived from the epistemic Global North to analyze the Indian context require reorientation. Concepts borrowed from a Eurocentric episteme can propel a logic and language that are often not aligned with a Global South context. Turning to the work of Gloria Anzaldúa (6), who introduced the concept of the Borderlands to signify not only demarcated geographic regions, but also the psychic, sexual, racial, and class dwelling places where bodies become marked, maligned, and marginalized, was a reference point. Anzaldúa (6) states that in contrast to persons of the center who – on account of their majoritarian positioning – wield social and cultural currency, border dwellers are seen by the center as “*[l]os atravesados*… the squint-eyed, the perverse, the queer, the troublesome, the mongrel, the mulato, the half-breed, the half-dead” (p. 3). Inhabiting the Borderlands gives rise to what Anzaldúa calls a *mestiza consciousness*: a distinctive awareness borne of the tensions and ambivalences of embodying multiple, often conflicting, identities. McLaren (7) described these “border identities” as those “intersubjective spaces of cultural translation”, spaces “where one can find an overlay of codes, a multiplicity of culturally inscribed subject positions, a displacement of normative reference codes, and a polyvalent assemblage of new cultural meanings”. Bakshi (8), in his commentary *On Decolonising Queerness,* observed how engaging Borderlands knowledge is a departure from received norms requires strenuous labor of not only undoing harmful forms of discourse on transgender communities and queerness across the globe, but also through a critical assessment of “what” non-normative sexualities and genders signified.

A challenge to the epistemic violence of Western epistemes (9) in “projects of contestation” ((10), p.100).

Picq and Tikuna (11) have proposed how the meanings of gender roles and sexual practices are cultural constructions that inevitably get lost when they are decontextualized in cultural (and linguistic) translation. The spectrum of Indigenous sexualities does not fit the confined Western registries of gender binaries, heterosexuality, or LGBT codification. It is not these idioms that are untranslatable, but rather the cultural and political fabric they represent. Indigenous sexualities defy contemporary LGBT and queer frameworks.

It is with these articulations of how knowledge is produced in and through a particular logic of language that drove our use of Ettinger’s (12) concept of wit(h)nessing, which re-situates affective, somatic, and multisensory knowing. Zembylas (13), p.120 cautioned that researching, analyzing, and reporting on subalternity requires attention to how it affects reproducing dominant social and political hierarchies and structures, forms of oppression, and exclusions.

Our objectives in this study are therefore to:

- To analyze and critique dominant biomedical discourses and the way in which dominant teaching practices within medical educational institutions create LGBTQIA+ identities using reductionist and interventionist paradigms.
- To investigate body pedagogies associated with LGBTQI+ identities.
- To conceptualize wit(h)nessing as an ethical and pedagogical means of engaging lived experience with healthcare education.

These may be viewed as a decolonizing pedagogy of disorientation, which has thematic resonance with Ahmed (14) understanding of queer orientation as a process to speak back to tokenistic diversity efforts that do not challenge the epistemological frameworks on which they are used and which create rather than ameliorate existing harm.

## Methodology

Situated with the epistemological foundation of standpoint theory (15) which claims that individuals from marginalized social positions have access to forms of knowledge that those who are in dominant social positions do not, we argue that individuals who are subordinate (in this case, those who are LBGTQIA+) develop forms of knowledge that draw upon psychological, social–emotional and medical well-being and safety as not differentially experienced phenomenon.

In this study, we draw upon Coffey (16) notion of Body Pedagogies to generate a prism from which to read the LGBTQI+ body as being actively re-produced through affective relations, rather than a passive object upon which social and cultural meanings are inscribed, and situate body pedagogies as resistance practices in health professions education in the Global South. Drawing on phenomenological and critical hermeneutic traditions (17–21), we aim to think beyond clinical categories, to reconsider how the body is regulated within various social and medical contexts (11, 22, 23). Deleuze has suggested that bodies ‘become’ through interactions and connections with other bodies, things, and the surrounding world; that the body is relational becoming “never separable from its relations with the world” [(18), p. 628].

Body pedagogies start from the premise that people who identify as LGBTQIA+ are not problems in need of solutions (45), but rather as holders of valid expertise based on their experience of navigating through spaces not designed for them, affording an epistemic advantage because they must learn about both the dominant social perspective and their own experience with oppression (which challenges the social narrative of the dominant group).

Pedagogical questions are never only pedagogical—they are also about power, care, and what kinds of lives become possible inside hegemonic institutions. For example, a trans woman who has spent the last several decades honing her ability to identify meaningful shifts in social space (e.g., by interpreting threat and possibility from subtle variances in reactions to her presence) has acquired a level of phenomenological insight that cannot be found in any medical textbook. Similarly, a lesbian couple raising children in an unrecognized family unit has developed innovative models of resilience, care networks, and alternative definitions of kinship within their community that have significantly impacted how society organizes itself. Situating body pedagogies as an educative agent and an affirmative mode of integrating Queer diversity, equity, and inclusion, we draw on Ettinger (12) notion of wit(h)nessing that situates relationality as an onto-epistemic entanglement as a counterbalance to the epistemicide of LGBTQI bodies. Ettinger (12) claims that affective, somatic, and multisensory encounters must be prioritized over purely visual or intellectual observation (in the pursuit of extracting scientific fact or evidence). The deep acknowledgment of human entanglement with others and the imperative to act with care create a space of wit(h)nessing that is larger than the attention given to one participating individual practitioner and can be used to analyze institutional pedagogy whose knowledge is recognized as curriculum, who’s body can be a teacher, and what does it mean for students to learn from a person rather than simply from learning about the subject matter.

The objectives of the study, as well as the conceptual framework, were utilized to develop the schedule for both the interviews and workshops. The first set of questions focused on curricular content, institutional norms, hidden curriculum, hierarchies, discrimination, and how LGBTQIA + identities are represented or pathologized in teaching and clinical scenarios. The second set of questions addressed the pedagogical values that inform the design of curricula; the educational roles of formal and informal learning contexts, and to what extent the diversity of bodies, identities Moreover, lived experiences are reflected in healthcare education curricula. In relation to the third objective, the questions invited participants to consider gaps, resistance, preparedness, community engagement and opportunities for the redesign of curricula in ways that promote more relational, inclusive, and ethically responsive forms of teaching and learning, consistent with the body pedagogies and the process of wit(h)nessing.

## Method

This study adopts a multi-method qualitative design and situates the everyday knowledges of LGBTQIA+ healthcare students and educators as epistemic sites that unsettle dominant biomedical discourses. Framing embodied experience as a relational becoming (18), we center non-normative bodies as pedagogical agents to counter LGBTQIA+_epistemicide in health professions education in the Global South (47).

### Research design

Data collection proceeded in two sequential phases. Phase 1, through interviews and participant observation, addressed curricular pathologization and colonial tensions. Phase 2 addressed wit(h)nessing as a pedagogy of queer disruption strategies. This design ensured theoretical coherence, with phase 1 establishing baseline inequities and phase 2 intervening pedagogically to generate transformative data.

### Participants and sampling

30 LGBTQIA+ students (MBBS *n* = 15, BSc Nursing *n* = 10, BPT *n* = 25) and 12 health professions educators/curriculum designers were purposively sampled from public and private institutions in Delhi-NCR and West Bengal. Sampling maximized variation across gender identity (cisgender, trans, non-binary), sexuality (gay, lesbian, bisexual, queer), program year (I-IV), and regional context, reflecting intersectional marginalization in India (24, 25). Snowball sampling enabled access to hard-to-reach trans and indigenous-identified participants (e.g., Hijra community affiliates). Inclusion criteria required direct experience of curricular/clinical erasure; heterosexual allies were included if they witnessed institutional bias.

### Phase 1: semi-structured Interviews and participant observation

Semi-structured interviews (45–60 min, *n* = 30) explored curricular normativity (default cis-men body as universal standard, LGBTQIA+ as pathological/at-risk), colonial tensions (Western diagnostics cs. Indigenous identities like Hijra/Chakka/Launda), clinical biopower (secondary violence, surveillance via forms/wards), epistemic authority (patient lived knowledge cs. Textbook dominance). Schedules used open-ended questions with probes (e.g., “How does the curriculum treat bodies outside the men, cisgender standard?”). Interviews were audio-recorded and transcribed verbatim.

The lead researcher conducted participant observation in naturalistic settings – lectures, ward rounds, skills labs – across five institutions. As a queer-identified observer, somatic responses (discomfort, tension) were logged as co-constitutive data, enacting wit(h)nessing methodologically (12). Field notes followed a protocol: verbal/non-verbal content, absences/silences, laughter/corrections, and institutional spatial dynamics (e.g., ward allocation). Observations captured informal curricula (jokes, mimicry), normalized beyond retrospective recall.

This phase responds to earlier research that has demonstrated that there is a pathological representation of LGBTQIA+ content in Indian MBBS programs (26), who provided evidence of this content being restricted to disease categories such as HIV risks and gender identity disorders. Sekoni et al. (27) stated that queer bodies are portrayed as either disease carriers or spectacles (see Table 1). The questions posed to the participants were explicitly designed to explore such gaps connected to the previous conversations about LGBTQIA+ exclusion, e.g., “When thinking about your approach to working with allied health students, what principles or value system influence how you develop your course or classes?”

**Table 1:** Orientating the research.

Domain	Sample interview questions
Curriculum and representation	Q. What is typically used to inform curriculum, such as syllabi, learning objectives, and assessments? Q. In addition to formal curriculum materials, what additional learning opportunities are available through clinical placements, ward visits, or other informal encounters?
Exclusion and institutional culture	Q. Can you describe the overall culture at your institutions regarding the conversations around gender, sexual orientation, and social identity in a healthcare-related context? Q. Based on what you have experienced, do you think faculty and students have difficulty addressing certain topics? If so, why do you believe this is the case?
Informal and embodied learning	Q. When LGBTQIA + members are referenced in your teaching materials, case studies, and clinical scenarios, how do you think they are represented? Q. Have you ever seen or do you hear from other students/faculty/staff that the textbooks used in the classes or courses provide a pathologizing or marginalizing view of LGBTQIA + populations?
Change and inclusion	Q. How would you design your curriculum differently if you had the authority to redesign your curriculum to ensure it is inclusive of all health and identity types? Q: Do you believe that there is a specific mandate or support role that regulatory agencies, e.g., the National Medical Commission or nursing councils, should have regarding LGBTQI + inclusive education?

### Wit(h)nessing workshops and embodied methods

According to Damery et al. (28) and Sekoni et al. (27), there is a significant dearth of health education research on LGBTQIA+ people in the Global South, particularly regarding community-oriented embodied forms of pedagogy. Wit(h)nessing workshops were created not exclusively for data but as a methodological challenge to evidence relating to the exclusion, by setting up opportunities for somatic and relational knowledge to be applied to test the framework. All participants attended 3 wit(h)nessing workshops, each 3 h long. Intervention was theoretically required: wit(h)nessing demands somatic familiarization before reflection (29). Post-workshop (1–2 weeks), follow-up interviews (30–45 min) explored wit(h)nessing enactment and queer disruption strategies. 20 participants maintained 4-week reflective journals, logging real-time instances. Observations continued (10 h) during workshops.

### Data analysis

Data generated underwent reflexive thematic analysis (30): (1) familiarisation, (2) inductive coding, (3) theme generation, (4) layer-mapping to wit(h)nessing, (5) review/reflexivity, (6) reporting. Somatic coding captured embodied data (e.g., “chest tightness”). Themes aligned with objectives: pathologization/risk, colonial gaps, wit(h)nessing layers, queer resilience.

## Analysis

Our analysis situated Ettinger (12) notion of wit(h)nessing as an ethical enactment that requires co-presence and somatic resonance prior to discursive reflection. Wit(h)nessing does not reject clinical rigor. However, by expanding the apparatuses used to generate clinical knowledge, it also expands a pedagogical architecture in which educators and teacher-patients intentionally create spaces for the student and living knowledge to interact. Oliver (31) has suggested wit(h)nessing is to be an eyewitness, to see, to watch, and to bear testimony.

Wit(h)nessing in blending being with knowing provides new tools with which to evaluate many aspects of the actions that have historically been excluded from the evaluation of the actions of clinicians themselves. These are the harms created/by clinicians during clinical encounters (specifically within the space where clinician-client encounters occur, in addition to the clinician’s notation of the encounter), as opposed to merely the client’s body on the examining table.

Wit(h)nessing is a way of staying with “the trouble of staying” (50) and in analysis requires the researcher to engage with the disarray and inconsistency of the material “data” rather than making premature determinations about how they should be categorized for analysis. The analysis of interview data from educators in both medicine and nursing reveals persistent barriers at the institutional, cultural, and pedagogical levels that impede the integration of LGBTQIA+ health into Indian health professions curricula, as well as aspirational pathways to reform these barriers. Examples of higher-order themes, such as “Institutional and Regulatory Barriers” and “Cultural and Historical Resistance,” demonstrate how regulatory gaps, hierarchical conservatism, moral/religious opposition, and misattributed colonial legacies create the “Curriculum of Absences” for gender-diverse content in favor of cis-normative priorities. Examples of “Pathologization and Risk Framing” and “Clinical and Informal Curriculum Gaps” illustrate the use of dehumanizing strategies to characterize MSM as disease vectors, use derogatory terms such as “chakka” within documents, and view transgender patients as spectacles; these strategies normalize both stigma and structural violence within both formal educational environments and clinical practice settings. Conversely, examples of “Calls for Transformative Change” indicate a desire for integrated curricula that challenge established assumptions, have queer-friendly faculty members, and promote the unsettling of the traditional methods used to develop faculty members with a focus on health equity. Collectively, these examples indicate a curricular structure that communicates to individuals as to whose bodies and lives are “valuable”; unless there is policy-driven reform grounded in participant values, this structure will continue to perpetuate systemic inequities.

### Institutional and regulatory barriers

Foucault (32) concept of hospitals as sites of biopower reveals how medical institutions function as mechanisms for regulating bodies through measurement, classification, and normalization, ultimately defining particular visions of social life. Participants acknowledged how, in clinical spaces, LGBTQ sexuality is positioned in relation to moral codes and rigid gender binary categories. The absence of an institutional LGBTQ affirming process, it was noted, has implications for patient experience at the point of care, with provider contempt in care encounters normalized.

Participants noted how:

*Registration forms force you into being men or women. If your Aadhaar says one thing and your body reads another, staff turn it into a public spectacle. (P3, MBBS-III).*

*Ward allocation is decided ad hoc. Sometimes trans women get pushed into men wards ‘for safety’, which means for staff convenience. (P11, MBBS-IV).*

*By the time the trans patient actually met the doctor, she had already been humiliated three times at registration and triage. (P7, MBBS-II).*

*Registration forms force you into being men or women. If your Aadhaar says one thing and your body reads another, staff turn it into a public spectacle. (P3, MBBS-III).*

*A gay man with anal pain was questioned like a criminal: ‘Why did you do such things?’ There was zero attempt to hide the judgment. (P12, B. Sc. Nursing-III).*

The structural entanglements of institutional and regulatory barriers in Indian health care domains re-established distinctions between “us” and “them,” “insiders” and “outsiders,” “health,” and “pathology.” The interviewees referenced affective dimensions of unbelonging; emotional and psychic alterity, and how abjection involved technologies of management and governance which reproduced stigmatizing discourse in the health care space. We now turn our attention to the factors that explain the persistent failure of health care providers’ efforts to create Queer-inclusive and welcoming spaces.

### Pathologization and risk framing

The prevalence of harms of exclusions and the confluences of cis-gendered and heteronormative models were noted to be intimately connected to corporeal taxonomies of identity. Corporeal taxonomies of identity are systems or classifications that categorize individuals based on physical characteristics. The labels or categories assigned to individuals based on gender, race, age, body type, and appearance provide a framework for understanding the organization of identities within health care domains and comprise a complex network of rules and meanings that shape perception, determining what we see and what we overlook, and can be considered. The participants commented on how.

*We hear about gay men only in the HIV class, as if that is their whole identity- carrier of infection, nothing else. (P3, MBBS-III).*

*The first time I saw the word ‘lesbian’ in our material, it was on a slide titled ‘sexual deviations’. I felt my stomach drop. (P7, MBBS-II).*

*“Trans people are introduced as a ‘challenging group to manage.’ That phrase says everything about how we are supposed to see them. (P10, B. Sc. Nursing- II).*

*When a trans patient leaves, staff mimic their walk. It becomes entertainment. You start to fear what they do when you walk out of the room.” (P7, MBBS-II).*

*A gay man with anal pain was questioned like a criminal: ‘Why did you do such things?’ There was zero attempt to hide the judgment.” (P12, B. Sc. Nursing-III).*

*Our community medicine slide showed hijras as a cartoonish group at a traffic signal. The class laughed. No one asked why that image was chosen.” (P7, MBBS-II).*

*One faculty used an old WHO document that still said ‘risk behavior of homosexuals’ and did not bother to correct the language. (P13, MBBS-III)*

*A surgery professor joked, ‘If you behave like this, we will send you to the hijra ward.’ The class roared; no one objected. (P6, MBBS-II)*

*When a trans patient leaves, staff mimic their walk. It becomes entertainment. You start to fear what they do when you walk out of the room. (P7, MBBS-II)*

*A senior passed us notes where ‘transsexualism’ was in the section on personality disorders. We are literally being trained with recycled violence. (P14, MBBS-I)*

When everyday mockery and body shaming go unchecked, it gives the green light for LGBTQIA+ people to navigate a healthcare system that pathologizes their existence and draws attention beyond individual participating practitioners to institutional pedagogy whose knowledge is recognized as curriculum.

### Clinical and informal curriculum gaps

Bodies marked by emblematic signifiers of difference are, we suggest, habitually located in the onto-epistemic creases of marginalization and are subject to a rhetoric of deficit as a regulatory presence. The participants commented that:

*LGBTQIA individuals were framed within ICD-10 codes for gender identity disorders.*

*In lectures, ‘hijra’ appears only on the HIV prevalence slide. Nothing about culture, history, or state violence – just prevalence. (P2, MBBS-II)*

*There is no lecture on queer people just coming with diabetes or back pain. We only appear as a risk factor or diagnostic label. (P15, BPT-III)*

*There are no sample cases where the patient has a same-sex partner. It is like those families simply do not exist in medicine. (P11, MBBS-IV)*

*I realized I have never seen a textbook image of a trans body that was not dissected into hormones and surgeries. (P8, Nursing-III)*

*Our training does not recognize that some of us are living the very realities they present as abstract ‘case scenarios.’ (P8, B. Sc. Nursing-III)*

Consensus, consultation, and cooperation are not neutral goods, and the authority of the medical center that renders refusal of the unthinkable is how Queer knowledge is dismissed as foreign to the clinical setting. Being seen involves a proto-ethics of response-ability and relationality beyond a clinical role. We now turn to what the interviewees had to say about disrupting the narrative of heteronormative authority in the clinical setting.

### Calls for transformative change

In the interviewee’s everyday worlds of clinical practice, what does it mean for students to learn from a person rather than simply from learning about the subject matter was queried?

*The teacher has never lived that reality. (P11, MBBS-IV).*

*Do not bury us in one psychiatry lecture. Integrate us into cardiology, surgery, pediatrics – everywhere, because we are everywhere. (P1, MBBS-II).*

*We need queer faculty… faculty development… lasting, uncomfortable, genuinely transformative engagement.*

*If a hijra says the medication is causing side effects, it is dismissed as ‘drama’. Their bodies are not trusted, even about their own pain. (P5, MBBS-III).*

## Wit(h)nessing

*I entered the ward with my clipboard and my checklist. A gay man in his late thirties had been admitted following a panic attack. My supervisor whispered to me that his ‘lifestyle’ was ‘part of the problem’. I wrote that down, but something in me resisted the notation. He was not a lifestyle. He was a man who had been outed to his family by the triage nurse. I put down my clipboard. I sat. I did not diagnose-I wit(h)nessed. That sitting, that pause was the most clinical thing I did all morning. It told me more about the ethics of care than any module had.* (MBBS-III, Participant 14, Women, She/Her)

The phrase “the most clinical thing I did this morning” needs to be said over and over again, because it does not represent a clinical competence retreat, but rather a reconceptualization of what that means; this pause creates an understanding of the situation that could not be achieved through a checklist, or an understanding that could be gleaned through the process of clinical notation.

According to Foucault (33), the “clinical gaze” transforms one’s status as a person into that of a “legible medical body” and, therefore, renders irrelevant the social and historical dimensions of the person’s situation in the process of producing knowledge about them; the physical tool that facilitates this transformation is a patterned alternative (the clipboard). Putting the clipboard down does not imply the abandonment of a clinical practice but rather temporarily suspending the clinical gaze’s colonization of the person with whom the clinician encounters and remaining present with the person prior to the gaze having transformed him/her.

Wit(h)nessing tbis interaction raises an urgent and compelling question about how the moment of resistance to erasure is possible and how it can be somatic rather than cognitive; sensed before cognitively conceptualized. Resistance to develop a clinical assessment describes a moment of spatial revision that does not result from categorically resisting a particular way of witnessing; rather, it is the act of changing their context by sitting next to their patient (rather than standing at their head). This change in behavior represents a small difference in the modeling they are given; it conveys an attitude of availability/horizontality to their patient that their patients observe and respond to.

The expression ‘sat with’ has a microcosm of the grammatical construction of wit(h)nessing; it remains unprocessed, uncategorized, and unintegrated into a previously existing clinical history. “I just ‘sat with’” implies a gift of trust being extended to the patient, one that goes beyond simply providing him with clinical information; it extends to social repercussions as well. The practitioner’s somatic and affective encounter with the patient as a queer-identifying clinician should be treated as something we get to listen to (as opposed to something to control) rather than being dismissed as ‘noise’. Their experience of sitting and being present in the ward exemplifies the extension of clinical epistemology into new, uncharted areas, rather than examples of deviation from that method of knowing about the body and performing clinical work. The clinician’s body (as somatic register), her “felt experiences of wrongness”, her embodiment of experiences endured by others, and her ability to be present in the moment to the level of encounter being experienced are components of the clinician’s body and mind as entangled instruments of knowledge production. These instruments provide information regarding the relational context of the institution in which such encounters occur, and the relational dynamics between the clinician and the client encountered (48). More broadly, the somatic perspective supports the notion that all clinical knowledge is produced by specific, contextualized practices and that these practices exhibit some degree of overlap in their production of clinical knowledge.

A nursing faculty member who invited a Hijra Activist to teach in her classroom challenged the standard curriculum, not simply adding diversity to it, but rather offering an onto-epistemic knowledge that the mandated curriculum does not allow for. That is a type of knowledge that is presential (exists in that space with the student) and not propositional (does not exist until it is documented), and gives the student (the student’s physical presence in the same space as) the person that the curriculum has intentionally deemed as non-existent, thus having a physical response to the confusion created by that new presence. They reported:

*When I brought a Hijra activist into the classroom as a co-teacher, my students were visibly unsettled. That unsettlement was the pedagogy. Their bodies were registering the collision between their training and a living, breathing epistemology that their textbooks had erased. That collision-that somatic friction-is where ethical education begins*. *I tell my students wit(h)nessing someone’s pain is not enough. You have to be with it, inside the discomfort of it* (P08, Nursing faculty, They/Them)

The Hijra activist in a classroom does not represent a marginalized community whose health care experiences should be known to students. The Hijra activist represents a type of knowledge that is embodied, immediate, and irreducible to any category supplied by textbooks; the mere presence creates a point of collision between the students’ trained perceptual framework and a reality that their framework was not built to handle.

A transgender men patient-teacher in another seminar room uses an expression to articulate their onto-epistemological stance that challenges the established conventions of clinical education.

*When I speak to these students, I watch their faces move through discomfort, recognition, and sometimes, if I am lucky, something that looks like accountability. I am not there to be their case study. I am there so they wit(h)ness what it costs to navigate a hospital corridor when every form asks for a gender that does not match your body. I am the pedagogy and in wit(h)nessing me, they are learning something no curriculum committee has yet approved, that I am the expert on my own life* (Trans man, P02, He/Him, Patient-Teacher Narrative)

“I embody Pedagogy” is such a precise and effective claim that we need to consider it for a while before continuing. The claim is not “I have relevant experiences that teach about curriculum” or “I am one case that helps develop students’ abilities to be culturally competent”; rather, I physically present myself in this classroom for all the reasons I have said, with my spoken and physical presence and all of the things associated with my body and how it costs to use it within healthcare institutions which are not able to name the forms of my body. This produces the knowledge or experience. My body demonstrates what a curriculum should look like, not what I will show students. The knowledge I bring does not supplement what students are gaining from their learning at this time; it existed before they fund that learning and is much more fundamental, and of a type for which no institutional committee has yet arrived at an administrative language for approval.

Until now, these accounts have focused on demonstrating the presence of wit(h)nessing through instances of one-off encounters (a ward round, a clinical consultation, a seminar room transformed by an individual whose existence was unplanned for by the curriculum). However, there is also a documented, much longer, more institutional-type account of when a person must also manage their own visibility before they can wit(h)ness in the institution that employs them and remain employed.

*I was the faculty member who raised questions about ‘comprehensive care’ and ‘patient diversity’ in language that gave no one cause to look at me too carefully. I had learned, the way one learns these things, that visibility in an institution is a resource to be managed, not a right to be exercised* (Medical Education Researcher, Gay Man, He/Him)

Enacting the dynamic of his own managed invisibility in the institution, a gay professor notes how surviving in the institution is contingent upon concealing his gay identity when initiating a motion to include LGBTQIA+ health topics in a curriculum review meeting. For the previous 11 years, these topics had been forced into optional elective courses where they had become institutionally invisible.

## Discussion

Scholars such as Nama et al. (23) and Damery et al. (28) have documented systemic deficits in LGBTQIA+ health training internationally and in healthcare education across the Global South. Specifically, in India, there is a notable lack of LGBTQIA+ curriculum content in medical education. Agarwal and Thiyam (26), in an article published in The Lancet Regional Health Southeast Asia, reported that throughout the MBBS undergraduate curriculum in India, LGBTQI+ health information is absent or referenced only in a pathological model. Their description of the National Medical Commission’s (NMC) recent curriculum revision (11) as the first major update in decades also indicates an absence of training incorporating queer affirmative perspectives. This finding directly correlates with the participant data presented in this report, in which LGBTQIA+ identities are represented in formal instructional materials as either a disease vector, a psychiatric diagnostic entity, or an exceptional clinical presentation (27, 34).

In this study, we have approached the issue through a distinctively situated lens, which generated the four broad themes of Institutional and Regulatory Barriers, Pathologization and Risk Framing, Clinical and Informal Curriculum Gaps, and Calls for Transformative Change.

The analyzed vignettes of the medical student who puts the clipboard down, the clinical student who “sits with” a patient’s gift of disclosure. The allied health student who identifies their somatic discomfort as clinical data provides a substantial analytic basis for a new conceptualization of what constitutes clinical knowledge. The nursing educator’s creation of a controlled disorientation in her classroom sets up a controlled disorientation for her students by bringing an external force into the educative space, creating discomfort and disrupting what they assumed to be ‘normal’. In all cases, the body/mind experience cannot be condensed into the standardized systems used in clinical training; rather, it is the resistance to this reduction that creates an ethical understanding. Inviting a Hijra activist to act as co-teacher did not add diversity to the existing curriculum but rather created ‘somatic friction’, which is the intentional pedagogical collision between trained perception and embodied knowledge that the curriculum has systematically excluded from consideration. The somatic element is consistent with Levinas (1969) ethics of the face, which defines an inalienable responsibility to the presence of the Other. However, this somatic aspect framed in an educationally actionable manner marks a shift from asking students “What do you observe?” to “What do you feel you must do?” It represents a relatively small change in how questions are posed to students. However, this shift demonstrates a substantial change in the ethical architecture of education by redefining students from epistemic subjects (who know something) to moral agents (who have an obligation to act).

This is a shift from existing theoretical frameworks of reflective practice in the health professions (49) from the cognitive domain to the somatic domain, providing empirical evidence that ethical re-orientation among healthcare practitioners can be initiated through the body rather than the mind. What the allied health student describes as “felt wrongness” reinforces the growing number of available resources on AOP (Anti-Oppression Pedagogy) in the field of health professions education (35). Smith et al. (36) scoping review published in Advances in Health Sciences Education indicates that AOP integrated into health professions curricula supports students’ critical reflection on the structures and systems of oppression that produce health inequities.

The power of exclusion that is exerted through medical institutions is productive rather than solely repressive; it does not just erase LGBTQIA+ identities, it constructs them through the identification of risk, deviance, and spectacle. Michael et al. (46) outlined the extent to which the non-inclusion of LGBTQIA+ topics in core curricula, rather than in optional modules, inevitably sends a structural message that some populations are the basis around which medical knowledge is constructed and that the health care of others is considered irrelevant. This distinction has implications that go beyond diversity as an enhancement to the curriculum. The findings of this study indicate that to successfully integrate LGBTQIA+ health into practice and policy, there must be a fundamental restructuring of the ways in which knowledge is validated (i.e., by whom knowledge will be validated, by whom their bodies will be used as part of the curriculum, and how various forms of knowing will be accepted by the medical/clinical profession). The findings of this study suggest that improving medical education in the Global South goes beyond changes to the materials taught. Participants’ recommendations for integrating queer material into all clinical areas, such as cardiology, surgery, and pediatrics, rather than only into a single module on either a psychiatric or community medicine basis, illustrate that there needs to be a profound structural redefinition of what constitutes clinical knowledge and, consequently, who holds that knowledge. Research on structured programs shows that even small, well-focused interventions can help improve students’ knowledge and awareness of LGBTQIA+ individuals as patients, specifically when grounded in intersectional barriers and social determinants (37–39).

## Conclusion

Homosexuality and gender diversity in quotidian Indian medical discourse call for cultural introspection (34); a rethinking to embrace diversity with compassion, and reclaim the inclusive legacies that colonialism erased. Vicars (40), p.206 has claimed “it is virtually impossible to extricate a single thread of how issues of power, transformation, action, agency exist independently of the epistemic Other” and whilst the colonizing violence of narratives of heteronormalcy has subjected the material reproduction of sexual subalternity to scripted ideological tropes.

The ongoing use of dehumanizing language (such as “chakka”) in official teaching resources, and the framing of MSM as vectors of disease. The spectacularization of transgender patients in clinical settings cannot be attributed to the individual bias of providers; rather, they are symptomatic of a “curriculum of absences,” which is a systematic erasure of the history and legacy of LGBTQIA+ people. Therefore, there is a lack of recognition of whose bodies can be considered legitimate objects of clinical understanding, which is conveyed most frequently through nonverbal means. This curriculum of absences is not reflective of an unbiased history, as Bajpayee (41) demonstrated how an ongoing negotiation of discourse that included both the imposition of regulatory and surveillance frameworks on non-normative bodies (42).

Diversity work is defined by Ahmed (43) as the work of bodies who exist in institutions where they must simultaneously enact the institution’s narrative of inclusion and absorb the costs of the structural reality that this narrative obscures. Situating the clinical gaze was a way to explain the normalizing actions of the medical community and classify bodies. In the Indian context, institutions act as sites of biopower as they regulate LGBTQIA+ bodies through hyper-pathologization and hyper-visibility.

Homosexuality and gender diversity in quotidian Indian medical discourse call for cultural introspection; a rethinking to embrace diversity with compassion, and reclaim the inclusive legacies that colonialism erased (34). Disclosure for many Indian LGBTQI+ individuals will remain deeply tied to a fear of rejection, both from society and from their heritage. The active inscription of language onto bodies, creating and constraining their meanings, is a product of wording that makes language become not merely a tool of communication but a regulatory force, shaping how queer bodies are read or erased. The genderqueer body ceases to speak itself because the structures of signification are already coded to interpret its emergence as excess, threat, or perversion of the coherence of normativity. The spectrum of Indian lived queer experience in medical health education demands a departure from a Western “default human” body that obscures the spectrum of Indigenous cultural and lived experience and obfuscates the complexity of geo-cultural and socio-political forces that, in the Indian context, compound discrimination and exclusion, even within spaces that are supposedly inclusive, becoming the accepted cultural responses of Otherness (25).

In this study, our narrating of genderqueer identities in Indian health care education demanded a departure from a Western imaginary of knowledge production. Western conceptions of gender and sexuality fall short of capturing the entwined and entanglements of caste and Queerness. The potential of body pedagogies to reclaim medical health education in the Global South from Western conceptions of gender and sexuality situates the entwined and entanglements of knowledges drawn from the embodied self. Wit(h)nessing makes visible opportunities to challenge the normative structures that create medical knowledge. Body pedagogies acknowledge that everyone involved in the health education experience (students, patients, instructors) has embodied knowledge that contributes to the learning experience (29). If healthcare students learn to reflect upon and utilize their own bodily responses (such as discomfort, disgust, and desire) to the hospital environment, their patients, and how particular bodies elicit emotional responses, then in their respective roles as institutional agents (44).

## Data Availability

The data analyzed in this study is subject to the following licenses/restrictions, data is still active. Requests to access these datasets should be directed to Shuvojitmoulik@gmail.com.
